# Report of Chairman of Section on Obstetrics and Gynecology*Read before the Central Texas Medical Association at Waco, July 10, 1900.

**Published:** 1900-12

**Authors:** A. R. Kuykendall

**Affiliations:** Morgan, Texas


For Texas Medical Journal.

### Report of Chairman of Section on Obstetrics and Gynecology.*

BY A. R. KUYKENDALL, M. D., MORGAN, TEXAS.

The medical profession should be proud of the progress made in the obstetric and gynecologic department.

From a study of the habits among primitive people, it would appear that had there not been a great revolution in the social and domestic affairs of the human family, little needful would be the demand for the obstetrician during the parturient period. Little do we know of diseases of women among the first inhabitants of our country. It is interesting, from a study of "Labor Among Primitive Peoples," as given by Engleman, to note the wonderful manifestation of the power of instinct. Can one imagine what would have been the result had God failed to endow man with animal instinct, which forms the connecting link to abstract thought?

The advance of civilization and the introduction of style and fashion have developed in the human family a deviation from original manners, and a pervertion of the modes of life. The indulgence of our tender female friends in these new styles of dress and habits of life has brought malformations and deformities both physical and psychologic.

The animal instinct so strongly relied upon by the females of our savage and semi-civilized races is lost in a degree to their educated sisters of today. And since the inhabitants of our country of today represent a "standard-bred grade of humanity," the fairest and angelic side of the family, those whose physical and moral culture should be the highest obtainable in view of her motherhood duties, are sadly neglected; and this neglect, coupled with the errors of dress and habit, enters as the predominant cause of abnormal and difficult labors, as well as the many aches and ills peculiar to the sex.

We may say, also, the instinct has become so perverted as to account for the numerous cases of neurotic manifestations, irritable temper, hysterical fits, mental horror—even temporary insanity, if *per chance* she becomes pregnant. We recall that among certain tribes of Indians the mother was instinctively assisted in her labor by compression. This assistance was given by a sister asso-

*Read before the Central Texas Medical Association at Waco, July 10, 1900.

ciate, who, with arms around waist, clasped hands over abdomen of patient and pulled down. It is with a blush of indignant censure, yet a feeling of pity for our girls and women, that we note our observation in that so many of the dear ones of today are adopting this method of squeezing the thing out of their bellies with laced corsets, and with many the effort at compression is begun as soon as she becomes cognizant of her condition.

That we have made great progress in obstetrics and gynecology goes without saying, and the very nature of things has demanded progress. The great minds of the profession have, in the past century, been greatly taxed in an effort to devise ways and measures of helping our deformed females to carry babies to maturity, and to safely bring them forth.

In no department of our profession is woman called on to appreciate her medical adviser and help more than in that of the obstetrician. As a rule, the family physician has the care of the pregnant woman from the time she conceives until after the lying-in period; hence, many are the importunities of the anxious mind of his female patient, and great are the opportunities for studying the demands and the devising of methods of safely tiding woman to her motherhood.

From a scientific point of view, the medical man of a progressive trend of mind can selfishly tip his hat to his fashionable female friends in grateful acknowledgment for this opportunity to develop and advance this interesting scientific field. Much reform, however, is needed in the manner of dress and in the development of the physical frame of our girls and young women, that they may become proportionately and adequately matured for the duties of procreation and the bringing forth of their young.

Therefore, it behooves and properly becomes the duty of the scientific medical man and family physician to vehemently insist and advise these reforms, thereby tending to restore nature's ideal womanhood, giving her beauty and tenderness that counterpart of strength and form so necessary to happiness and longevity.

The popular idea of today that woman is only to be pretty and shapely and adorned with flashy dress and catchy style—suited only as a toy; a plaything in the arms of man simply to gratify passion—is erroneous, and a veritable curse to humanity, and defeats the high aim of the Almighty when he found that 'twas not good for man to be alone.

Natural philosophy teaches us that in electricity the like poles repel and the unlike poles attract each other; and that when the positive and negative elements approach each other the spark will fly. Observation and experience, gentlemen, in human affairs of life recalls that this physical law is imitated in the emotional laws of the animal. It is an admitted fact that there is an irresistable attraction for the opposite sex, and when the male and female are brought together the spark is sure to fly. Man, however, is prone to become overawed by the glitter and sparkle of the flash, and falls victim of a desire to gratify passion, rather than a conscientious meditation and study of the results. Indeed, there are too many marriages between disproportionate men and women; hence, difficult labors, resulting in wilted, faded, exhausted woman, unfit and incapable for the duties of motherhood. Too often do we see a small, delicate woman—low and slender in stature—the wife of a tall, raw-boned man of 200 pounds. The union of these disproportionate men and women should be discouraged, for such offers many chances for fatal accidents during labor, or injuries to mother that will leave her a hopeless invalid. These unfortunate circumstances, as well as other causes, are responsible for the many ills and aches peculiar to the female. I know of no other field within the bounds of medicine and surgery which offers the doctor greater scientific study and work than the care and management of diseases of women. Not many years ago poor, helpless woman was the victim of the patent medicine vendor, or when peradventure she appealed to her family physician she was drenched with tincture or fluid extracts, or made to swallow pills and potions. The vagina was then a too convenient a cavity into which to squirt nasty washes with glass squirt-guns. Too often was it the receptacle for a medicated tubule. The demand which this subject has made upon the medical mind for more scientific management of the troubles of the gentler sex has caused to be placed at our command the result of study and genius,—the many devices for examination, the assistance of mature thought and experience making our diagnosis more correct, thereby assuring a more satisfactory line of treatment.

With these hints, gentlemen, I give over to you whose study has been deeper and whose experience much wider than the writer's, this very interesting and quite important subject, and beg of you a thorough and intelligent discussion.

